# ILA Guidelines for Sustainable Natural Resources Management for Development

**DOI:** 10.1007/s40802-021-00190-x

**Published:** 2021-08-30

**Authors:** Marie-Claire Cordonier Segger, Nico J. Schrijver

**Affiliations:** 1 Leiden University, Leiden, The Netherlands; 2 Board of Editors NILR, The Hague, The Netherlands; 3 University of Waterloo, Waterloo, Canada; 4 Cambridge University, Cambridge, UK

## Introductory Note

On 13 December 2020, the 79th Conference of the International Law Association (ILA) adopted by consensus the ‘ILA Guidelines for Sustainable Natural Resources Management for Development’ (Resolution 2020/4). This resolution was passed at the conclusion of its Kyoto session, which in view of COVID-19 constraints took place virtually for the first time in history. The ILA Sustainable Natural Resources Guidelines had been prepared by the ILA Committee on the Role of International Law in the Sustainable Natural Resources Management for Development, in which Professor Marie-Claire Cordonier Segger (Canada) and Professor Nico Schrijver (the Netherlands) served as Rapporteur and Chairperson, respectively. This Committee was established in November 2012, building on a distinguished history of ILA enquiry into international law on sustainable development and with a traditional focus on the developmental dimensions. The ILA Committee engaged 49 legal expert members from over 30 countries from six continents, and over eight years met in collaborative working sessions in Leiden (2015), Montreal (2017), Cambridge (2018), Athens (2019) and Lugano (2020, in hybrid form).^1^

Global concern about sustainable natural resource management is increasing significantly. In response, the field of international law on sustainable development has been rapidly evolving. The Committee adopted a broad notion of ‘natural resources’. Systematic deliberations focused on: (1) global natural resources that have been recognised as common concerns or the common heritage of humankind, such as celestial bodies, the atmosphere and a stable climate system, biological diversity and ecological systems, and the ocean and its mineral and living resources; (2) regional and transboundary natural resources of global importance such as forests and landscapes, rivers and freshwater ecosystems, and migratory species; and (3) national natural resources of global relevance such as forests and landscapes, land and soil, mineral commodities, including precious minerals, and sustainable energy. The experts on the ILA Committee considered instances in which commitments to the sustainable management of natural resources for development have been enshrined in international treaty law, reflected in the practices of States and international organizations and in the decisions of international courts and tribunals, and increasingly operationalized in international ‘soft law’ goals, standards and guidelines in relation to different resources, taking into consideration selected national level developments where relevant.

Three principal pillars formed the Work Programme of this ILA Committee:

(i) The study and analysis of the contents, legal status and application of the principles and rules of international law related to the sustainable management of natural resources at the international and national levels, as well as an assessment of the practice of States, and international organizations in this field;

(ii) An examination of the relationship between the evolving international law in the field of sustainable development and the principle of the sustainable use of natural resources, particularly analysis of:

- the status of the obligation of States to use natural resources in a manner that is sustainable, including issues such as the obligation to undertake impact assessments of plans and projects that might affect sustainable development, transboundary resources management, the sharing of resources in the world interest and taking into account the interests and needs of future generations;
- innovative instruments to support sustainable use of natural resources and their status and implementation in international and national law, including measures within regional trade and investment agreements and multilateral economic treaties;
- the relationship between natural resource management and the enjoyment of human and peoples’ rights;
- decisions of international courts and tribunals on matters related to natural resource management.

(iii) The study of national and international approaches to the regulation of natural resources in developing countries and the impact of such approaches on the sustainable use of natural resources and on the evolution of international law in this field.

In essence, the ILA Committee’s work has demonstrated that the world’s natural resources, once seen as national and subject to permanent sovereignty, are now increasingly recognized as eliciting common global, regional, transboundary and national concerns, thereby challenging international law to provide more coherent and effective cooperative regimes for sustainable management. Tensions can exist with regard to certain nationally-based resources, however myriad rules and standards now define, guide and direct State practice, providing a roadmap for the progressive development of international law on the sustainable management of natural resources for development.

In the view of the ILA Committee, international law has the potential to shape the principles, regulatory frameworks, institutions, standards and incentives for natural resource management on multiple local, national, regional and global levels. After a thorough review, the Committee’s findings suggest that international law both reflects and also serves as a critical catalyst for the design, adoption, and implementation of sustainable natural resource management and resolution of disputes in relation to the use of natural resources. Global natural resources which have been recognised as common concerns or the common heritage of humankind crucially require increased collective commitment and compliance with the international law and practices reflected in the newly adopted ILA Sustainable Natural Resources Guidelines. Regional and transboundary natural resources of global importance are also greatly at risk, and their sustainability depends on the crafting, implementation and enforcement of effective and collaborative international legal regimes. Further, the sustainable management of national natural resources of global relevance can be greatly facilitated by international law. Indeed, international law and non-binding international and national instruments such as standards and guidelines function as a baseline for States and other actors involved in natural resource management, shaping operating environments in which sustainable development will either be fostered, or frustrated.

In assessing the role of international law in this context, it is crucial to take into account how concepts of sovereignty and territory are evolving to accommodate new scientific understanding of interrelated ecological systems and conditions, whereby notions of custodial sovereignty may offer useful insights. The Committee’s work has established that sovereignty, of key importance to international law from its inception, is becoming both more fluid and more qualified in the face of shared responsibilities for the sustainable use of transboundary, regional, and global international natural resources, and collaborative regimes for management. The Committee has identified tensions in the context of nationally-based natural resources, and has considered how international legal regimes may offer options to reconcile key concerns, avoiding or reducing the potential for conflict between and within States over resource use, as well as defusing potential clashes between resource conservation and exploitation goals.

Natural resources are also essential to advance nearly all 17 Sustainable Development Goals, and many of their 169 targets, from poverty elimination, to ending hunger, to access to water and energy, to combating climate change and promoting peace, justice and security.^2^ The Committee noted that international natural resources management systems could make a vital contribution to the achievement of the Sustainable Development Goals and the 2030 Agenda worldwide, but also that how these regimes are governed will be crucial for their implementation and enforcement. The Committee’s work highlights a number of governance mechanisms, ranging from formalized compliance mechanisms to informal industrial and sectoral oversight procedures, which implement essential tools for sustainable natural resources management and the sustainable use of natural resources.

These Guidelines reflect both established international law, including *lex lata* rules of treaty law that are binding on the Parties as well as customary rules, and also many norms that are still *lex ferenda*, with a view to future directions in law-making.

The Guidelines are organized in three parts. The First Part (I) presents, sector by sector in a non-exhaustive survey, certain guidelines for the sustainable management of global, regional, transboundary and national natural resources, covering: (1) global natural resources such as celestial bodies, the atmosphere and a stable climate system, biological diversity and ecological systems, and the ocean and its mineral and living resources; (2) regional and transboundary natural resources of global importance such as forests and landscapes, rivers and freshwater ecosystems, and migratory species; and (3) national natural resources of global relevance such as forests and landscapes, land and soil, mineral commodities, including precious minerals, and sustainable energy. The Second Part (II) addresses trends and innovations in international legal instruments and approaches in sustainable natural resources management for development, with a non-exhaustive selection covering trends from: (1) human rights approaches; (2) economic instruments; (3) environment and sustainable development cooperation including scientific collaboration, financing mechanisms, monitoring, reporting and verification, and public participation and access to information and justice; and (4) peacebuilding and post-conflict instruments, including secure land and water access. This Part also covers (5) innovative techniques and requirements in international instruments on the sustainable management of natural resources for development, with special attention to: (a) sustainable resources management through transparency and stakeholder engagement, (b) equitable benefit-sharing from sustainable natural resources management, (c) legal indicators of effectiveness for sustainable natural resources management, and (d) control of illicit flows for sustainable natural resources management. The Part further covers a brief, non-exhaustive update on (6) sustainable natural resources management in international dispute settlement. Finally, in the Third Part (III), explanatory notes are provided for the interpretation and application of the 2020 ILA Guidelines on the Role of International Law in Sustainable Natural Resources Management for Development.

These ILA Kyoto Sustainable Natural Resources Guidelines build on and contribute important insights to earlier key normative standards of the ILA, including the ILA Seoul Declaration on the Progressive Development of Principles of Public International Law relating to a New International Economic Order, published in 33 *NILR* (1986), pp. 326–333 and the ILA New Delhi Declaration of Principles of International Law Relating to Sustainable Development, published in 49 *NILR* (2002), pp. 299–305.

## Footnotes

1 The final report of the Committee as well as its previous biannual reports can be consulted under ‘Committees’ at www.ila-hq.org. The Chair and Rapporteur gratefully recognize the contributions of the experts from the Committee, particularly the editorial group including Prof. Ilaria Espa (Italy), Dr. Alexandra Harrington (Colombia), Cairo Robb (UK), Dr. Fabiano de Andrade Correa (Brazil), Prof. Emmanuella Doussis (Greece) and Dr. Nadia Sanchez Castillo-Winckels (Chile/the Netherlands), as well as from the team of dedicated legal researchers from the University of Victoria, Canada, including Ellen Campbell and Courtenay Jacklin, and from the University of Cambridge, including Freedom-Kai Phillips, Chantalle Byron, Yanela Ntloko, Ra’chel Thorpe-Blair, Fabiana Piccoli Araujo Sanchez, David Gayle, Tejas Rao, Beth Pasfield and Timothy Arvan.

2 See *Transforming Our World: The 2030 Agenda for Sustainable Development*, UN Doc. A/RES/70/1, 25 September 2015, which includes the 17 global Sustainable Development Goals (SDGs).

